# Gene expression patterns during adaptation of a helminth parasite to different environmental niches

**DOI:** 10.1186/gb-2007-8-4-r65

**Published:** 2007-04-24

**Authors:** Emmitt R Jolly, Chen-Shan Chin, Steve Miller, Mahmoud M Bahgat, KC Lim, Joseph DeRisi, James H McKerrow

**Affiliations:** 1 California Institute for Quantitative Biomedical Research (QB3) of the University of California, San Francisco, 4th Street, San Francisco, CA 94158 USA; 2 Theraputic Chemistry Department, Infectious Diseases and Immunology Laboratory, the Road to Nobel Project, the National Research Center, Dokki, 12311 Cairo, Egypt

## Abstract

**Background:**

Schistosome bloodflukes are complex trematodes responsible for 200 million cases of schistosomiasis worldwide. Their life cycle is characterized by a series of remarkable morphological and biochemical transitions between an invertebrate host, an aquatic environment, and a mammalian host. We report a global transcriptional analysis of how this parasite alters gene regulation to adapt to three distinct environments.

**Results:**

Utilizing a genomic microarray made of 12,000 45-50-mer oligonucleotides based on expressed sequence tags, three different developmental stages of the schistosome parasite were analyzed by pair-wise comparisons of transcript hybridization signals. This analysis resulted in the identification of 1,154 developmentally enriched transcripts.

**Conclusion:**

This study expands the repertoire of schistosome genes analyzed for stage-specific expression to over 70% of the predicted genome. Among the new associations identified are the roles of robust protein synthesis and programmed cell death in development of cercariae in the sporocyst stages, the relative paucity of cercarial gene expression outside of energy production, and the remarkable diversity of adult gene expression programs that reflect adaptation to the host bloodstream and an average lifespan that may approach 10 years.

## Abstract 2

Using a genomic microarray, gene expression at three different developmental stages of the schistosome parasite were analyzed, resulting in the identification of 1154 developmentally enriched transcripts.

## Background

**Figure 1:**
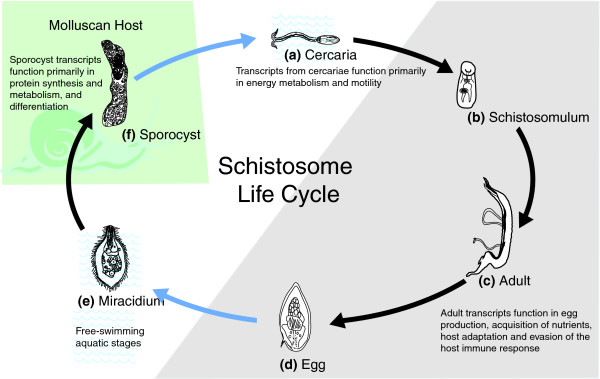
The schistosome life cycle is complex, with morphologically distinct stages occupying several ecological niches. Infective cercariae **(a) **swim in fresh water to find and infect a mammalian host. After host invasion, cercariae transform into schistosomula **(b) **and adapt to survival in the host bloodstream. The schistosomula mature into adult male or female schistosomes **(c)**, which pair and produce eggs **(d)**. Eggs are excreted from the host. In fresh water, the eggs hatch into miracidia **(e)**, which infect a snail host and develop into sporocysts **(f)**. Daughter sporocysts generate infectious cercariae, completing the life cycle.

Schistosomiasis is a chronic debilitating parasitic disease affecting some 200 million people across 74 countries within Africa, Asia, the Middle East and South America. In terms of public health and socio-economic impact, it ranks second only to malaria among parasitic diseases [1,2]. The causative agents of schistosomiasis are schistosome bloodflukes, multicellular trematodes whose life cycle is characterized by a series of striking morphological and biochemical transitions between an intermediate host snail in an aquatic environment, two free-swimming aquatic larval forms, and a warm-blooded mammalian host (Figure 1). As such, the schistosome represents an ideal but challenging biological system in which to identify programs of gene regulation that have evolved to facilitate adaptation of metazoa to different biological microenvironments.

In the intermediate host aquatic snail, sac-like clusters of differentiating larvae called daughter sporocysts give rise to large numbers of free-swimming aquatic cercariae. Cercariae navigate their environment by a side to side motion of their tails and find the mammalian host through chemical and environmental signals [3-6]. Upon entering the mammalian host, the tail is detached, and an early vascular stage called the schistosomulum rapidly readjusts to the new host environment. During this time, the schistosomulum undergoes changes in basic metabolism and surface properties [7]. The schistosomulum navigates the vascular system, including transiting through at least two capillary plexuses. Between 14 and 28 days, the organisms undergo rapid growth and differentiation to adult male and female forms in specific regions of the host vasculature. Male and female worms form mating-pairs and the female produces hundreds to thousands of eggs per day, depending on the species of schistosome. The eggs, in turn, pass out of the host via feces or urine, and hatch in fresh water to yet another morphologically distinct form, the miracidium. This larval form moves through fresh water by means of numerous cilia, and invades the soft parts of the intermediate host snail, transforming into the mother sporocyst. The cycle is complete as the mother sporocyst produces daughter sporocysts, which serve as the developmental stratum for production of cercariae.

Efforts to reduce schistosome prevalence have included combinations of sanitation, health education, snail control, better diagnosis and chemotherapy [8]. Control of parasitemia has relied primarily on praziquantel, an effective chemotherapeutic drug that has been in use for over 20 years. Recurring morbidity and evidence of emerging resistance to praziquantel in endemic countries emphasizes the need for both an effective vaccine and new chemotherapy [9-11]. Several potential vaccine candidates have been identified [12,13], but to date none is sufficiently effective for practical use. Given the complexity of the schistosome lifecycle and its ability to evade the immune system, a more comprehensive approach to understanding the molecular biology and biochemistry of schistosomes is essential to rationally identify and develop potential vaccine candidates or new drugs.

The approximately 270 Mb genome of *Schistosoma mansoni *[14] is organized into 8 chromosomes [15], including the heterologous female pair WZ [16]. It is estimated to have 30% GC content [17]. Currently, the *S. mansoni *genome is thought to contain some 14,000 predicted genes [18,19]. Until recently, there have been relatively few attempts to analyze the functional genomics of schistosomes due to limited genomic sequence information. Identification of genes expressed in a sex-specific manner by adult worms was made using either a 576, 7,335, or 4,608 oligonucleotide cDNA based microarray system [20-22] and an analysis of genes associated with pairing of adult male and female worms was addressed [23]. The transition between miracidium and mother sporocyst was also analyzed using the same 7,335 component oligonucleotide array noted above [24]. Comparisons of adult transcripts of two schistosome species, and comparison of lung stage versus experimentally produced schistosomula, were carried out using a larger array [7,25]. A comparative study between the *S. japonicum *proteome and transcriptome was also undertaken [26].

Now that the genome is nearing completion, we present a comprehensive analysis of gene expression during three of the major stages of *S. mansoni *development: daughter sporocyst, cercaria, and adult. These stages represent key transitions from intermediate host snail to aquatic environment to mammalian host. We designed a 12,000 oligonucleotide microarray chip made of 45-50-mer oligonucleotides and we analyzed expression of a majority of the predicted 14,000 *S. mansoni *genes under very stringent conditions. While the present annotation of the genome does not distinguish gene insert orientation, we found that over 9,700 of the oligonucleotides printed yielded clear transcriptional signals in at least one parasite stage. We have examined pair-wise differential expression at each stage and identify those genes whose RNA expression profile is conserved or differentially expressed across developmental stages. Noteworthy gene clusters that support previous hypotheses or provide new insights into the responses of the parasite to environmental transitions during the lifecycle are identified. Highly expressed transcripts in sporocysts include those involved in general protein synthesis and quality control, consistent with the function of sporocysts in production of large numbers of cercariae. Cercarial transcripts are dominated by genes involved in mitochondrial function, supporting the energy production necessary for cercarial swimming. Adult worms express a diverse pattern of transcripts necessary for egg production, energy metabolism, immune evasion and physiological maintenance of a relatively long-lived organism.

## Results

### The 12,000 element schistosome array

The microarray used in this study contained 12,000 individual 45-50-mer oligonucleotides based on 11,998 tentative consensus sequences (TCs), as documented by the *Schistosoma mansoni *Genome Index maintained at The Institute for Genomic Research (TIGR) [27]. TCs are created by virtual assemblage of full or partial cDNA sequences into transcripts. Spliced forms of genes are listed separately. Of the 12,912 sequences provided by TIGR, 11,998 TCs were chosen based on the maximum size of the TC available. Thus, this microarray contains a majority of known sequences available in the *S. mansoni *genome. There have been concerns raised about the orientation of inserts in the current annotation of the TIGR assemblage; however, we found that >9,700 of the 12,000 oligonucleotides printed gave some transcript signals in one or another parasite stage by type II analysis (see Materials and methods) and, as detailed below, many key gene programs could be identified and correlated with environmental transitions.

### The microarray chip is specific for *S. mansoni *transcripts

Extraction of RNA from schistosome daughter sporocysts involves excision of the entire snail hepatopancreas, and co-lysis of the snail and schistosome material. To control for any background hybridization of snail-specific material (to our *S. mansoni *cDNA-based 12,000-oligonucleotide microarray), we hybridized hepatopancreas RNA from infected snails containing daughter sporocysts versus uninfected snail hepatopancreas RNA across four different chips. With the exception of two genes, we found that intermediate host snail RNA was not detected (data not shown). Even for these two genes (TC8129, similar to beta-1 4-galactosyltransferase, and TC6896, similar to Unknown), hybridization signals were not seen on every run and were detected only by type II analysis. In summary, no significant contribution of snail RNA to the hybridization analysis was found. Daughter sporocysts contain cercariae at various stages of differentiation. Nevertheless, the transcriptome profile of this stage in the snail differed significantly from that of mature aquatic cercariae.

We also found that microarray samples and chips were reproducible and correlative. There was an average Pierson correlation coefficient (log ratio of medians) of 0.7 for biological replicates and 0.96 for technical replicates. Dye switch experiments showed that data differences were not due to dye-labeling bias (Additional data file 1).

### *S. mansoni *transcriptome overview

**Figure 2:**
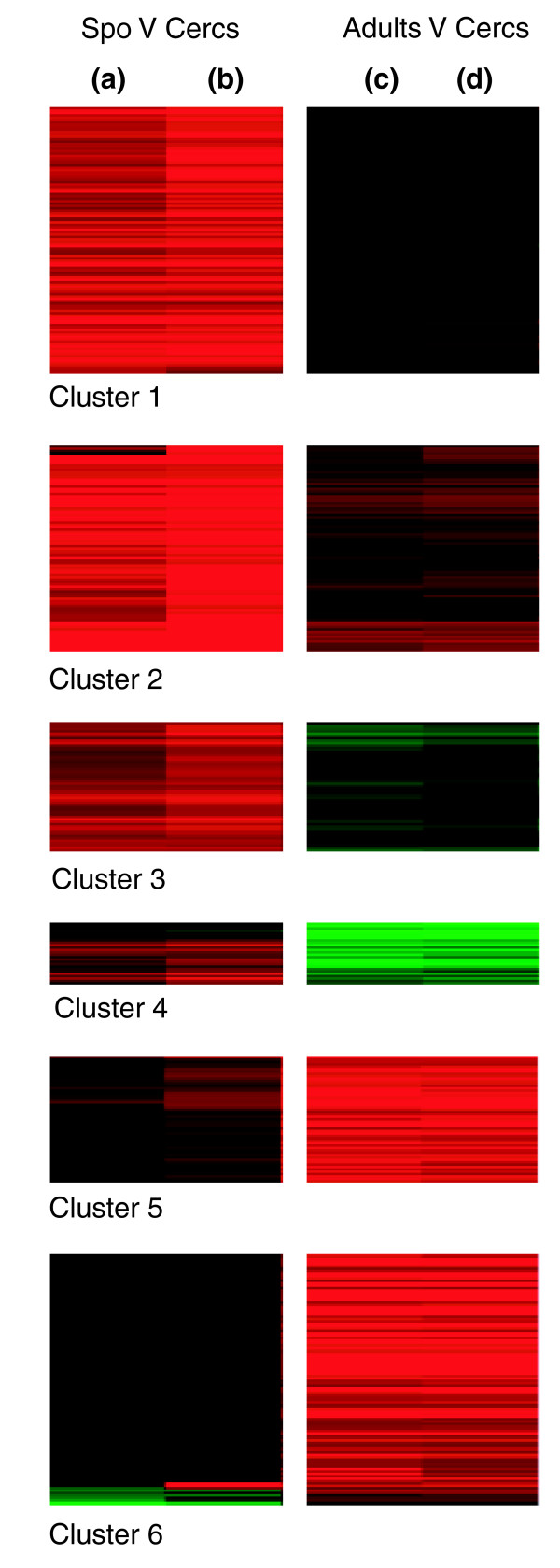
Schistosome transcripts are developmentally regulated and divided into six major gene clusters. Genes are differentially expressed in sporocyst/cercarial pair-wise comparisons (columns **(a) **and **(b)**, 'Spo v Cercs') or adult/cercarial pair-wise comparisons (columns **(c) **and **(d)**, 'Adults v Cercs'). Genes are highly enriched (three-fold) in sporocysts relative to cercariae (clusters 1-3), in cercariae relative to adults (cluster 3 and 4), and in adults relative to cercariae (clusters 2, 5, and 6).

**Figure 3:**
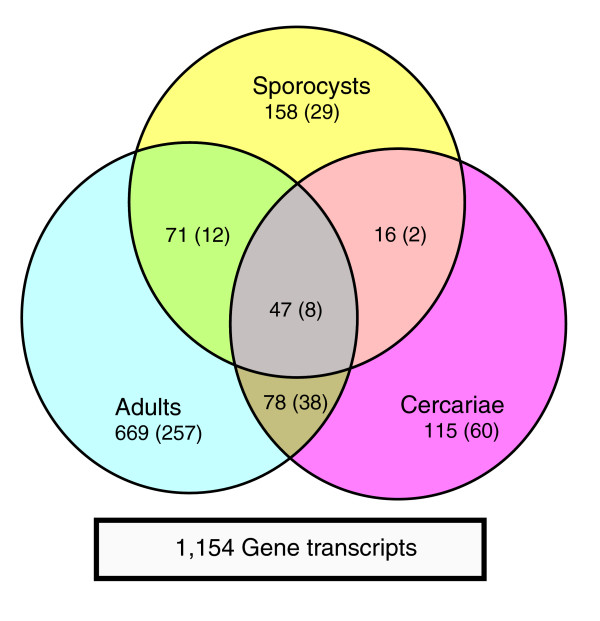
Venn diagram of known gene transcripts enriched (1.5-fold) during schistosome development. The number of unknown genes associated with each section is in parentheses.

This clustering analysis includes 431 genes analyzed by pair-wise comparison with at Examination of all developmental stages showed a transcript pattern specific to each. Figure 2 (Table 1) is a clustering analysis of the major transcripts identified in which duplicates of sporocyst (Cy5) were compared to cercariae (Cy3). For example, cluster 1 represents transcripts that are upregulated in the daughter sporocyst stage but not significantly in adults or cercariae. Cluster 2 shows transcripts up-regulated in both sporocysts and adults relative to cercariae. Clusters 3 and 4 show transcripts enriched in cercariae relative to adults. Clusters 5 and 6 show transcripts that are upregulated in adult worms but are either relatively low or not enriched in cercariae or sporocysts. least a 3-fold difference in transcript level between developmental stages. Overall, we identified enrichment of 1,154 genes during the schistosome developmental life cycle, the largest proportion of which is expressed in adult worms (type II analysis). Of the 1,154 genes, 406 represent genes of unknown function (Figure 3).

### Validation of microarray data by real time PCR

**Figure 4:**
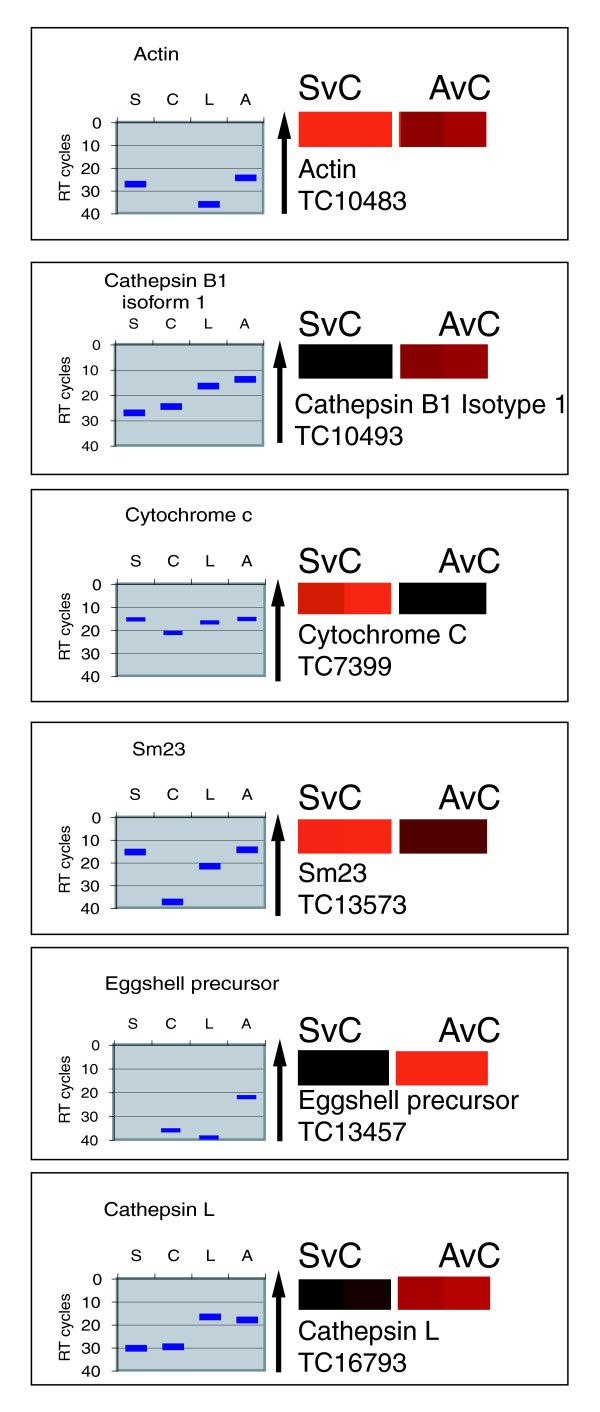
Real time PCR analysis of select transcripts across four developmental stages: sporocysts (S), cercariae (C), schistosomula (L), and adult worms (A). The upward arrow indicates transcript level where fewer RT cycles are indicative of an increase in transcripts relative to a cytochrome c internal control. The microarray ratio of sporocyst to cercaria (SvC) and adult to cercaria (AvC) is designated 'fold change' and is in the same format as Figure 2. Cathepsin L numbers are from type II analysis.

To test the validity of the microarray results, we performed a quantitative analysis study on the expression levels of several parasite genes by real-time PCR. Among the transcripts analyzed were actin, cathepsin B, cytochrome C, eggshell protein precursor, Sm23 and cathepsin L. Gene-specific primers were designed to validate the gene expression profile for sporocyst, cercariae, and adult developmental stages (Additional data file 2). To ascertain gene expression transitions between cercariae and adults, we also analyzed transcript levels in 24-hour schistosomula, the stage of schistosomal development between cercarial penetration and adult worms. We found that the expression of genes analyzed by RT-PCR correlates with our microarray analysis in 10 of 11 cases (Figure 4).

### Genes expressed in daughter sporocysts reflect production of cercariae

The daughter sporocyst stage resides in the intermediate host snail. The key biological function of this stage is to support the differentiation and development of large numbers of cercariae, the aquatic larval stage that will initiate invasion of the mammalian host. The function of this stage in generating cercariae is underscored by the fact that many of the most highly expressed transcripts are gene products that function in general protein synthesis (40S and 60S ribosomal subunits, elongation factor) and post-translational protein folding and fidelity (chaperones, ubiquitin). Furthermore, many of the proteins that will be utilized by cercariae in the initial stages of host skin invasion are also produced in this stage, including cercarial elastase (aka cercarial protease), cercarial muscle proteins (actin, dynein light chain isoforms), and calcium binding proteins that are also abundant in the proteome of cercarial secretions [28].

As would be expected from the large-scale differentiation of cercariae within the daughter sporocyst, transcription factors such as the Y-box binding protein and stathmin, a phosphoryl protein involved in vertebrate growth and regulation of differentiation [29] are highly expressed. Sporocysts express a cathepsin L-like cysteine protease homolog (TC9217) that is not significantly enriched in adults (Figure 4f). In adults a cathepsin L is gut localized and functions in digestion [30]. The lack of significant enrichment of other gut-specific genes in sporocysts, such as those encoding cathepsins B, B1, and C, and the unique profile of the cathepsin-like protease homolog, suggests that this protease isoform may function differently in the daughter sporocyst, possibly in the generation of cercarial progeny. In *Caenorhabditis elegans*, a cathepsin L is essential for embryogenesis and development [31].

### Genes expressed in cercariae reflect energy production and motility

Cercariae are a relatively short-lived 'transitional' stage that are released from snails into fresh water and must swim to find, and ultimately invade, a mammalian host. Cercariae will not survive if they fail to enter the mammalian host before energy sources are exhausted. Consistent with this concept of the biological function of cercariae, transcripts that are upregulated are primarily factors necessary to sustain swimming behavior and invasion (Figure 2, clusters 3 and 4). The highest expressed transcripts are genes involved in mitochondrial function or energy metabolism. These include NADH dehydrogenase and its various subunits, cytochrome C and its homologues, and ATP/ADP carrier proteins. Other transcripts highly expressed in cercariae include structural and motility genes like actin and fibrillin and transcripts coding for a protease that plays a role in host invasion, cercarial elastase [32]. In general, fewer transcripts are found in cercariae relative to other stages.

### Genes expressed in adult worms

Compared to sporocysts and cercariae, the most abundant transcripts in adult worms (male and female worm pairs) are significantly more diverse. This reflects the fact that adult worms must evade the mammalian host immune system, maintain motility, acquire and metabolize a variety of nutrients, form mating pairs, and produce large numbers of eggs. In parallel with these functions, highly abundant transcripts in adult worms include a group of genes involved in protein degradation in the gut (cathepsin B, cathepsin L), egg production, and oxidative stress responses [33-36]. Genes involved in gonadal differentiation for both male and female worms, as well as genes coding for proteins for egg-associated proteins are readily identified. These include the anti-mullerian hormone receptor for males, and members of the RXR family and Smad family, which are cell signaling pathways previously associated with function of female gonads [37,38]. Highly expressed egg transcripts include eggshell protein precursor, major egg antigen, and several homologues of these proteins.

Perhaps most striking is the abundance of transcripts in adult worms from genes coding for surface proteins, including nutrient transporters such as the glucose transporter and a number of surface proteins of unknown function previously identified in research projects aimed at subunit vaccine development. These include Sm23 [39-45], integral membrane protein 25 [46], Sm14 [47,48], and 26 and 28 glutathione S-transferases [49-52].

## Discussion

The stage-specific transcriptome of *S. mansoni *provides an informatics foundation for the study of parasite gene regulation and a correlative for proteomic studies. This microarray study validates and extends observations made with a 7,335-oligonucleotide array from previously available expressed sequence tags (ESTs) [53], and correlates well with comparative analyses between the transcriptome and proteome of adult *Schistosoma japonicum and S. mansoni *[26]. With 12,000 oligonucleotides, a more complete picture of gene programs that mark transitions between key schistosome stages in distinct environmental niches is now apparent. The entire schistosome genome is estimated to have 14,000 functional genes. While it has been noted that the TIGR annotation to date has not distinguished the orientation of the inserts sequenced, we found that 9,700 of the 12,000 genes analyzed on this array gave clear positive signals for at least one stage. More importantly, many genes could be functionally annotated and gene programs correlating with environmental transitions of the helminth parasite discovered or validated.

One of the most remarkable aspects of the schistosome parasite is the dramatic morphological change that takes place between life cycle stages (Figure 1). These morphological changes parallel transition of the parasite between three markedly different environments. The daughter sporocyst stage is the late developmental stage in the intermediate host snail - a mollusk of fresh water habitats. Motile cercariae emerge from the snail and must navigate an aquatic environment to find the mammalian host. Following entry into the skin of the mammalian host, the parasite must now adapt to a different osmotic environment and a warm-blooded host. Finally, within the mammalian host, two distinct sexual stages develop and eggs are subsequently produced.

Array analysis comparing daughter sporocysts to the cercarial stage highlighted gene programs responsible for supporting larval development. The sporocyst stage is a very efficient protein synthesis factory. This correlates with the need for mass production of cercariae and the availability of nutrients from an intimate relationship with the snail host. In keeping with the complex morphological differentiation of cercariae with distinct body parts and 'organs' from an embryonic cell mass, daughter sporocyst transcripts included genes involved not only in general protein synthesis such as ribosomal and heat shock genes, but also transcripts annotated to function in programmed cell death (TC18024) and ubiquitination, a process key to morphological differentiation.

In Figure 2, cluster 2, daughter sporocysts share with adult worms a previously unrecognized requirement for transcriptional upregulation of antioxidant genes such as glutathione-S-transferase, thioredoxin, and thioredoxin peroxidase. This may reflect oxidative stress generated within the immediate snail host, or storage of these proteins in developing cercariae, so that they are available in the earliest stages of mammalian host invasion. Utilizing a 7,335 oligonucleotide array, Vermeire *et al*. [24] documented gene expression patterns between miracidia, the aquatic stage that invades the snail host, and the mother sporocyst stage, which is the initial stage following invasion of the snail host. As is the case for daughter sporocysts analyzed in our study, they also found upregulation of several genes involved in protein synthesis, the redox pathway, and proteolysis. This suggests that these gene programs are initiated in mother sporocysts following entry of miracidia into the snail, and sustained throughout the daughter sporocyst stage until mature cercariae leave the snail.

Transition to the cercarial stage is marked by a reduction in transcript level of a large number of genes as seen in clusters 1 and 2 of Figure 2. We found 116 genes whose abundance is reduced in cercariae by 3-fold relative to sporocysts (type II analysis where sporocyst intensity units are set >3,000). In comparison to other stages, the cercariae are less transcriptionally active. Fewer than 7 genes are upregulated more than 2-fold relative to adults and only 34 are upregulated when compared pair-wise to adult transcripts.

The cercariae express a cluster of genes consistent with the energy required to move rapidly through water in search of a mammalian host. These include genes functioning in ATP production and utilization, presumably for muscle function and swimming behavior. Consistent with previous Northern blot analyses and biochemical studies, much of the repertoire of proteins that cercariae use to invade the skin of their host, as well as structural proteins, have already been produced during cercarial development in the daughter sporocyst stage within the nutrient rich intermediate host snail (Figure 2a-d). Cercariae do express caspases and related cell death programs, which may be required for morphological remodeling during transition into the schistosomulum stage. Cercariae detach their motile tail and surface glycocalyx shortly after entry into the mammalian host. Large gland structures (acetabular glands) producing invasive proteases involute within 48-72 hours of invasion.

Residence of adult schistosomes in the mammalian host bloodstream is supported by expression of gene families that respond to oxidative stress and genes involved in adjustment and adaptation to a new osmotic environment (aquaporin) [54-56]. Adult worms have a major requirement to digest blood-proteins and acquire other nutrients (cathepsin B, glucose transporter) [33,35,57]. Schistosomes differentiate into male and female worms and expression of an anti-mullerian hormone receptor [58,59] is likely related to sexual differentiation. Female parasites produce hundreds to thousands of eggs per day as reflected in the upregulation of egg shell proteins. Recent analysis of gene expression in earlier intravascular stages can now be compared with the adult (versus cercariae) transcriptome presented here. Using a cDNA array, Dillon *et al*. [60] equated seven-day cultured schistosomula with lung schistosomula and compared gene expression across life stages. Chai *et al*. [7] utilized schistosomula directly obtained from the lungs of infected mice, and compared transcripts expressed to adult worms, cercariae, and newly transformed schistosomula. By and large, the genes we found expressed in adult parasites versus cercariae were similar to those observed in the comparison of lung worms versus cercariae. These included nutrient acquisition genes, such as those encoding the glucose transporter and the proteolytic cathepsins. The genes down-regulated in adult worms relative to lung schistosomula include several we found expressed in cercariae or daughter sporocysts, including those encoding the anti-inflammatory protein Sm16 and paramyosin.

The intravascular stages of schistosome parasites have a complex and highly adapted relationship with the mammalian host [61,62]. To support this relationship, intravascular stages upregulate surface proteins or receptors, some of which have homology to mammalian receptors and/or factors involved in signaling cascades (Additional data file 6). These include the anti-Mullerian hormone type II receptor [63] and the thyroid receptor interacting protein [64].

Developmental regulation of cell number and type by programmed cell death appears to be an important function in all stages of schistosome development analyzed, as it is in the nematode *C. elegans *[65]. Homologues for genes involved in programmed cell death include TC11294 and the DAP-1 homolog TC18024 (type II analysis).

Since the initial stages of the genome analysis of schistosomes, it has been clear that transposon-like sequences are common. Efforts to exploit these elements for genetic manipulation are ongoing [66,67]. It is noteworthy that one transposon (TC17720) is more abundantly expressed in the sporocyst than in any other developmental stage. It is also expressed in adults, albeit at low levels, as corroborated in the study by Gobert *et al*. [25]. There is also another retroelement (TC7011) highly enriched in adult worms.

This expanded array data set, focusing on life cycle stage transitions, should aid in current attempts to develop transfection and gene knockout studies for schistosomes by identifying those genes that are stage-specific versus others shared among different developmental states. For example, the gene encoding cytochrome C is expressed at comparable levels in all stages studied. In contrast, genes such as those encoding the cercarial elastase in the sporocyst stage, or eggshell components in the adult female stage, represent potential models for identifying and characterizing key spatial and temporal promoter elements, and ultimately molecular mechanisms of gene regulation. Fitzpatrick *et al*. and Moertel *et al*. also have studied gender-specific gene transcription in *S. manson*i using a 7,335 oligonucleotide array [53] and a 22,575 combined *S. japonicum *and *S. mansoni *array [68]. The results reported here for genes that can be attributed to either male or female worms largely validate these studies.

Analysis of the stage-specific transcriptional program of schistosomes also helps to validate and underscore differences in the levels of gene products noted in proteomic studies of schistosome life cycle stages. The levels of many gene products identified by proteomic analysis parallel transcription levels in this microarray analysis. In contrast to protozoan parasites like Leishmania [69], this may indicate that less post-transcriptional regulation operates in schistosomes. Having transcriptome data for three major stages, combined with proteomic data, should now facilitate a more focused analysis to determine to what extent RNA stability or other post-transcriptional mechanisms play a role in schistosome gene regulation. It was noted from proteome analysis that many genes are expressed across stages and these were, therefore, seen as less attractive vaccine targets [26,70]. The identification of several stage-specific patterns of expression in this present study should help to redirect efforts aimed at finding the most logical candidates for a subunit vaccine, and also identify new targets to explore for drug therapy.

## Conclusion

We show that the daughter sporocyst stage in the intermediate host snail functions primarily to support the development of invasive larvae (cercariae) by up-regulating expression of genes involved in protein synthesis, cellular differentiation, and programmed cell death. Many of the major structural and functional components of cercariae, utilized to later invade the mammalian host, are expressed and packaged prior to larval release from the snail. The aquatic cercariae themselves are less transcriptionally active than other stages, with an emphasis on production of proteins involved in energy metabolism and motility. This is in keeping with the function of these larvae in swimming from snail to mammalian host. Finally, adult parasites, which have adapted to survival in the mammalian host bloodstream, have a complex transcriptional program that supports adaptation to a new host temperature and chemical environment, evasion of the host immune response, acquisition of nutrients, and production of eggs for transmission to a new host.

## Materials and methods

All experiments were performed with *S. mansoni *of the NMRI Puerto Rican strain maintained routinely through *Biomphalaria glabrata *snails and Syrian golden hamsters as previously detailed [71].

### Schistosome stage collection

Daughter sporocysts were collected by dissection of whole hepatopancreas from six-week old infected *B. glabrata *snails, during maturation of daughter sporocysts and prior to cercarial release. Uninfected snail hepatopancreas was also collected as a control. Cercariae were collected in distilled water from infected *B. glabrata *snails using the light induction method as previously described [71,72]. Following exposure to light for 2 hours, 50-60 snails shed about 200-300 cercariae/snail. Several collections were pooled and used for RNA extraction. Twenty-four hour schistosomula were mechanically transformed as previously described [73]. Adult worms were recovered from the mesenteric veins by portal perfusion as previously described [74].

### RNA extractions

Total RNA was extracted from all biological samples for Combimatrix microarray analysis. Multiple uninfected and infected hepatopancreas, cercariae, and adult worms were homogenized in 1 ml Trizol (Invitrogen, Carlsbad, CA, USA) and RNA was extracted using the standard Invitrogen protocol. RNA was eluted in deionized water, quantified, and checked for RNA quality by UV spectrometry and agarose gel analysis. Total RNA (15 μg) for individual stages (coupled to Cy3 or Cy5) was hybridized to Combimatrix custom array microarray chips, as described in detail below (Combimatrix Corporation, Mukilteo, WA, USA).

### *S. mansoni *DNA oligonucleotide probe design

All probes were designed using the Combimatrix software CombiMatrix Automated Probe-design Suite (PDS), based on 11,997 TC groups from sequences at TIGR. TCs represent a virtual assemblage of ESTs. Of the 12,717 TIGR TC groups, 11,998 were used for the 12,000 spot array based on size of the TC source cDNA. Those with the smallest TC source cDNA sequences were excluded. The 12,000 sequences were loaded into the Combimatrix Custom Array content probe array system for probe design, one of which was spotted in triplicate using different oligonucleotide designs constructed by hand. The probe design system took each TC sequence and designed 45-50-mer probes to be unique to each gene with predictable thermodynamic behaviors.

### Microarray hybridization and data analysis

Combimatrix Custom Array microarray chip hybridizations were stringently performed in duplicate or triplicate. Hybridizations (3× SSC, 0.025 M HEPES, 0.28 μg/μl polyA, 0.05% SDS) occurred at 63°C for 18 hours to maintain specific binding efficacy. DNA microarray chips were scanned using an Axon4000B scanner at 5 μ resolution and 100% laser power and the images were analyzed with GenPix Pro 4 (Molecular Devices, Sunnyvale, CA, USA). Microarray data were stored in the NOMAD microarray database [78] and normalized by a global normalization using unflagged features with a regression correlation coefficient ≥0.75 and median intensity value >0 as previously described [75]. Data were clustered using Cluster 3.0 [76] and visualized in Java TreeView version 1.0.11 [77]. Clustering was done as below: all data were log transformed and filtered for the presence of 100% of genes and a standard deviation value of log_2 _1.5 or 3-fold gene expression, followed by correlation uncentered clustering of genes and arrays by complete linkage analysis. A second and less stringent analysis was repeated using the same methodology, except clustering was done with a standard deviation value of log_2 _0.6, or 1.5-fold gene expression (Additional data file 3). Each Combimatrix array has 12,500 spots; 148 are empty controls (no oligonucleotides) and 352 represent various small oligonucleotide controls. For a less sophisticated analysis of gene expression, on or off, each channel (635 and 532) was treated independently. The data were reanalyzed and normalized as described above. The average median value of the 148 empty spots for each chip was used as background and subtracted from the initial intensity unit value to produce a 'normalized value'. Spots from the 'normalized value' (IU) were retained as expressed genes. We refer to this type of analysis as type II analysis. To compare all three stages, the average median of all cercariae median values was used as a standard (Additional data file 4). Transcripts with an IU >2,000 are defined as enhanced.

### RT-PCR

A total of 1 μg parasite RNA from each stage was used to prepare double stranded cDNA using SuperScript™ II reverse transcriptase (Invitrogen) in the presence of oligo dT. Quantitative PCR (qPCR) experiments were repeated in duplicate using cytochrome c as an internal control gene. The relative quantification of our genes of interest was carried out by mixing cDNA from different parasite stages with SYBR Green PCR master mix in 96 well plates, which was then incubated in the Applied Biosystems (Foster City, CA, USA) 7500 Real-Time PCR System. The program included an initial melting phase for 2 minutes at 50°C, denaturation and hot start for 10 minutes at 95°C, followed by 40 amplification cycles (95°C, 15 s; 60°C, 1 minutes). The sequences of the primers and names of the studied genes are given in Additional data file 2.

## Additional data files

The following additional data are available with the online version of this paper. Additional data file 1 is an analysis of Cy3 and Cy5 dye-switch experiments comparing sporocyst to cercariae, and adults to cercariae. Additional data file 2 is a table of primer sequences used for real-time PCR. Additional data file 3 is a table listing transcripts enriched at least 1.5-fold relative to cercariae in adults or sporocysts. Additional data file 4 lists all genetic data analyzed by type II analysis. Additional data file 5 is a table listing the 431 genes highly enriched from the clustering analysis shown in Figure 2. Additional data file 6 is a table listing *S. mansoni *receptor-like proteins and log_2 _gene expression ratios. Additional data file 7 lists all oligonucleotide sequences used for the schistosome array. Additional data file 8 is a key to compare the schistosome TC sequence to the updated TIGR schistosome gene index (Version 6).

## Supplementary Material

Additional data file 1 To rule out dye specific effects, we performed dye-switch experiments for stage-specific comparisons. We plotted the log-ratio of the median (Cy5 signal versus Cy3 signal) from one chip verses the other dye-switched chip. Here we use a color scale to represent the density of each data point plotted. Most data points are accumulated at the red regions. If gene expression differences are small or subtle between two stages (**(a) **sporocyst versus cercariae and **(b) **adults versus cercariae), we expect their signals would center around the origin and be randomly distributed (red regions). For genes that are highly expressed, the signals would be high in one chip but low on the other chip. The scatter plots show the expected strong negative correlation between the dye-switched chips. The genes in the adult and sporocyst stages that are highly expressed are not artifacts due to the choice of dye. Click here for file

Additional data file 2 Primer sequences used for real-time PCR analysis Click here for file

Additional data file 3 TC names, annotation and relative ratio of sporocyst to cercariae and adult to cercariae are seen in columns A-F in duplicate. The background was removed from all samples manually as described in type II analysis after normalization in NOMAD, and analyzed using Gen Cluster 3.0 where samples were log transformed and screened to exclude samples with a deviation less than 0.6. All ratios are listed as log2 values. Click here for file

Additional data file 4 The data presented are not ratios, but are averages of raw expression data of each stage independently, where 65,367 is the highest value (we consider values <500 as background) Click here for file

Additional data file 5 All 431 genes highly enriched from the clustering analysis shown in Figure 2 Click here for file

Additional data file 6 Transcript comparisons are between sporocysts (Sp) and cercariae (Ce), and adult worms (Ad) and cercariae Click here for file

Additional data file 7 All oligonucleotide sequences used for the schistosome array Click here for file

Additional data file 8 Following experiments done within this paper, the TIGR database containing TC sequences was updated and TC names were changed and expanded. Here is a key mapping TC sequences used in this paper to the new database [78]. Click here for file

## Figures and Tables

**Table 1:** Some of the major transcripts from clustering analysis of the microarray data

Transcript	Description
**Cluster 1: highly enriched in sporocysts**	
TC16551	Similar to 90 kDa heat shock protein
TC13362	Homologue to 40S ribosomal protein S14
TC16641	Homologue to elongation factor 1-alpha
TC13505	Homologue to AUT1
TC10547	Homologue to ribosomal protein S8
TC17388	Similar to ribosomal protein S15a
TC7475	Similar to 60S ribosomal protein L8
TC11590	Ubiquitin/ribosomal fusion protein
TC7403	Similar to 60S ribosomal protein L30
TC9217	Weakly similar to cathepsin L-like cysteine proteinase
TC17720	Similar to putative high mobility group-like nuclear protein 2
TC11170	Similar to Usmg5 protein
	
**Cluster 2: highly enriched in sporocysts and enriched in adults**	
TC10486	Glutathione S-transferase 28 kDa (SM28 antigen)
TC11169	Similar to Usmg5 protein
TC13573	23 kDa integral membrane protein (SM23)
TC7377	Similar to Y-box binding protein
TC16738	14-3-3 protein homolog 1
TC6863	Homologue to 14-3-3 protein homolog 1
TC8189	Similar to dynein 8 kDa light chain flagellar outer arm
TC13511	Homologue to AUT1
TC13698	40S rRNA protein homolog
TC16537	Heat shock 70 kDa homolog protein (HSP70) (major surface antigen
TC10528	Probable dynein light chain (T-cell-stimulating antigen SM10)
TC11430	Thioredoxin
TC10691	Thioredoxin peroxidase
TC16806	Similar to NADH dehydrogenase 6
TC7615	Similar to ATP synthase lipid-binding protein-like protein
TC11552	Homologue to cytochrome b
	
**Cluster 3: highly enriched in sporocysts and enriched in cercariae**	
TC13775	Antigen SM21.7
TC13518	Homologue to NADH dehydrogenase subunit
TC17477	Homologue to NADH dehydrogenase subunit 2
TC17193	Similar to NADH dehydrogenase subunit 4
TC13722	Homologue to NADH dehydrogenase subunit 5
TC16807	Homologue to NADH dehydrogenase 6
TC7310	Homologue to cytochrome c oxidase subunit 1
TC14303	Homologue to ATPase subunit 6
TC16782	Enolase
TC16557	Myosin heavy chain - fluke
TC10480	Elastase
TC8910	Tubulin beta-2 chain.
TC17016	Stathmin-like protein
TC7850	Similar to actin-filament fragmenting protein
TC7158	Similar to fibrillarin
	
**Cluster 4: highly enriched in sporocysts and cercariae**	
TC13565	Myosin light chain
TC16878	Similar to glycerol-3-phosphate dehydrogenase [NAD+] cytoplasmic
TC17004	Homologue to cytochrome c
TC16870	ATP:guanidino kinase SMC74 (EC 2.7.3.-; ATP:guanidino phosphotransferase)
TC17192	Homologue to NADH dehydrogenase subunit 4
	
**Cluster 5: enriched in sporocysts and highly enriched in adults**	
TC16575	Major egg antigen
TC8654	Similar to anti-Mullerian hormone type II receptor precursor
TC13882	Similar to 26S proteasome regulatory subunit
	
**Cluster 6: highly enriched in adults**	
TC10493	Cathepsin B1 isotype 1
TC13457	Eggshell protein precursor
TC13487	Homologue to eggshell protein precursor
TC13492	Homologue to eggshell precursor protein
TC8804	Weakly similar to retinoid X receptor RXR-2
TC12198	Similar to mucin-like protein
TC7011	Similar to putative retroelement
TC16781	Extracellular superoxide dismutase [Cu-Zn] precursor (EC 1.15.1.1; EC-SOD)
TC10637	Weakly similar to aquaporin 9 (small solute channel 1)
TC9163	Similar to serine/threonine-protein kinase unc-51 (EC 2.7.1.-; (uncoordinated protein 5)
TC7005	Similar to putative senescence-associated protein

Transcripts enriched in specific schistosome stages are organized into six major clusters. For an updated list of the latest version of TC sequences see Additional data file 8.
