# Supplementary material for: Gene expression patterns during adaptation of a helminth parasite to different environmental niches
Source: Genome Biol. 2007 Apr 24;8(4):R65. doi: 10.1186/gb-2007-8-4-r65 (PMC1896014; doi:10.1186/gb-2007-8-4-r65)
Supplement: Additional data file 2 — Primer sequences used for real-time PCR analysis [file gb-2007-8-4-r65-S2.pdf]

Primer sequences used for real-time PCR

Gene	Forward oligo	Reverse oligo
Actin 1	5' -TGAGCGATTCAGATGTCCAG	5' -CTTCTGCATACGGTCAGCAA
Cathepsin B1-1	5' -ACTTGGTGGGCACGCTATAC	5' -TAATTCGACCGGCTGTTACC
Cytochrome C	5' -AGTTATGCGGTGTGGGTCAT	5' -TGCTCGAGTCAAAGGCCTAC
Egg shell protein	5' -ACTGCAACCTCCACCATAGC	5' -ATCGCCATAACCGCTATCAC
Sm23	5' -GGTGATTGTTGCCTGTGTTG	5' -GGAAAACAGAACGGGATTGA
Egg shell protein	5' -ACTGCAACCTCCACCATAGC	5' -ATCGCCATAACCGCTATCAC
Cathepsin L	5' -GTTGCTATCGCCCAACACTT	5' -TCCTCCCAGTCCATATCACA
