# Supplementary material for: Gene expression patterns during adaptation of a helminth parasite to different environmental niches
Source: Genome Biol. 2007 Apr 24;8(4):R65. doi: 10.1186/gb-2007-8-4-r65 (PMC1896014; doi:10.1186/gb-2007-8-4-r65)
Supplement: Additional data file 5 — All 431 genes highly enriched from the clustering analysis shown in Figure 2 [file gb-2007-8-4-r65-S5.pdf]

CLUSTER 1

TC14426	"weakly similar to autoantigen {Rhipicephalus appendiculatus}, partial (29%) "
TC16551	"similar to 90-kDa heat shock protein {Sus scrofa}, partial (34%) "
TC10537	"homologue to similar to GenBank Accession Number L24368 ribosomal protein S4 in Gallus gallus, complete"
TC11203	"weakly similar to CG6090-PA {Drosophila melanogaster}, partial (62%) "
TC10531	"similar to Probable dynein light chain 1 cytoplasmic. {Caenorhabditis elegans}, partial (95%) "
TC6866	"similar to similar to GenBank Accession Number U54584 histone H1 in Schistosoma mansoni {Schistosoma japonicum}, partial (81%) "
TC16809	"homologue to similar to HMG1-like protein - fruit fly {Schistosoma japonicum}, complete"
TC17950	"weakly similar to nucleolar protein family A member 2 {Branchiostoma belcheri}, partial (34%)
TC10532	"similar to Probable dynein light chain 1 cytoplasmic. {Caenorhabditis elegans}, partial (66%) "
TC10986	"similar to 40S ribosomal protein S17. [Human], partial (78%) "
TC9217	"weakly similar to cathepsin L-like cysteine proteinase {Tenebrio molitor}, partial (36%) "
TC12156	
TC12464	
TC15675	"homologue to hypothetical protein JC8.7 - Caenorhabditis elegans, partial (3%) "
TC16530	
TC17720	"similar to putative high mobility group-like nuclear protein 2 {Echinococcus multilocularis}, partial (87%) "
TC13362	"homologue to 40S ribosomal protein S14. {Podocoryne carnea}, partial (82%) "
TC18225	"similar to Threonyl-tRNA synthetase cytoplasmic (EC 6.1.1.3) (Threonine--tRNA ligase) (ThrRS)., partial (29%) "
TC13769	"weakly similar to Similar to chromosome 1 open reading frame 6 {Xenopus laevis}, partial (29%) "
TC17617	
TC15994	"weakly similar to Unknown (protein for IMAGE:3139787) {Homo sapiens}, partial (12%) "
TC10676	"homologue to NADH dehydrogenase subunit 3 {Schistosoma mansoni}, complete"
TC16641	"homologue to elongation factor 1-alpha {Schistosoma mansoni}, partial (26%) "
TC13505	"homologue to AUT1 {Schistosoma mansoni}, complete"
TC6879	
TC7399	"homologue to cytochrome c oxidase subunit 2 {Schistosoma mansoni}, complete"
TC10437	"protein disulfide-isomerase homolog precursor - fluke (Schistosoma mansoni), complete"
TC16813	"calmodulin {Caenorhabditis elegans}, complete"
TC8967	"homologue to unnamed protein product {Mus musculus}, partial (2%) "
TC14370	
TC8464	"similar to ribosomal protein L29 {Sus scrofa}, partial (55%) "
TC10545	"homologue to similar to NM_080743 serine-arginine repressor protein (35 kDa) in Homo sapiens, partial (97%) "
TC11522	
TC16682	"similar to Stress-70 protein mitochondrial precursor (75 kDa glucose regulated protein) (GRP 75), partial (89%) "
TC16569	
TC18752	
TC17205	"weakly similar to G2/mitotic-specific cyclin 3. [Baker's yeast], partial (12%) "
TC7433	
TC10394	"weakly similar to troponin I {Mizuhopecten yessoensis}, partial (10%) "
TC10701	"similar to C. elegans RPS-26 protein (corresponding sequence F39B2.6) {Caenorhabditis elegans}, partial (50%) "
TC17063	"similar to Unknown (protein for MGC:56007) {Danio rerio}, partial (6%) "
TC16381	
TC16859	"similar to ADP ATP carrier protein (ADP/ATP translocase) (Adenine nucleotide translocator) (ANT)., partial (51%) "
TC18051	"weakly similar to tetraspanin TE736 {Schistosoma japonicum}, partial (16%) "
TC16555	"weakly similar to ORF 73 contains large complex repeat CR 73 {Human herpesvirus 8}, partial (4%) "
TC7697	"weakly similar to BH3184~unknown conserved protein {Bacillus halodurans}, partial (23%) "
TC10538	"homologue to similar to GenBank Accession Number L24368 ribosomal protein S4 in Gallus gallus, partial (55%) "
TC9225	
TC8781	"weakly similar to Unknown (protein for MGC:27818) {Mus musculus}, partial (44%) "
TC10488	"similar to NM_079796 Ribonuclear protein at 97D in Drosophila melanogaster {Schistosoma japonicum}, partial (39%) "
TC13660	"homologue to similar to GenBank Accession Number BC016558 ribosomal protein L23a {Schistosoma japonicum}, partial (90%) "
TC16795	
TC10606	"similar to UNKNOWN PROTEIN {Lactococcus lactis subsp. lactis}, partial (1%) "
TC16850	"similar to 40S ribosomal protein S13. [Pig], partial (48%) "
TC16572	
TC8719	"similar to cytochrome c oxidase subunit I {Roboastra europaea}, partial (46%) "
TC15835	
TC13786	"similar to no similar protein found {Silene latifolia subsp. alba}, partial (7%) "
TC8344	
TC10515	
TC15564	"similar to histone H2A - spoonworm (Urechis caupo), partial (92%) "
TC16964	"weakly similar to Peripheral-type benzodiazepine receptor (PBR) (PKBS) (Mitochondrial benzodiazepine receptor)., partial (21%) "
TC17224	"weakly similar to hypothetical protein ZK783.2 - Caenorhabditis elegans, partial (6%) "
TC7508	"weakly similar to unnamed protein product {Mus musculus}, partial (63%) "
TC17223	"weakly similar to hypothetical protein ZK783.2 - Caenorhabditis elegans, partial (6%) "
TC19626	"weakly similar to cytochrome c oxidase subunit III {Roboastra europaea}, partial (52%) "

TC6887	"similar to gag-pol polyprotein {Clonorchis sinensis}, partial (3%) "
TC13374	"similar to 40S ribosomal protein {Perinereis aibuhitensis}, partial (74%) "
TC17248	
TC16678	"Glyceraldehyde 3-phosphate dehydrogenase (EC 1.2.1.12) (GAPDH) (Major larval surface antigen) (P-37), complete"
TC16546	"Heat shock 70 kDa homolog protein (HSP70) (Major surface antigen). [Blood fluke], partial (17%) "
TC6865	"homologue to cellodextrinase C - Pseudomonas fluorescens, partial (2%) "
TC7896	
TC10354	"similar to similar to GenBank Accession Number M94390 HEXBP DNA binding protein in Leishmania major, partial (77%) "
TC10536	"similar to ribosomal protein L4 cytosolic [validated] - rat, partial (50%) "
TC10527	"homologue to dynein light chain 2 {Mus musculus}, complete"
TC7569	"T-complex protein 1 alpha subunit (TCP-1-alpha) (CCT-alpha). [Blood fluke], complete"
TC11197	"similar to Probable microsomal signal peptidase 25 kDa subunit (EC 3.4.-.-) (SPase 25 kDa subunit) (SPC25)., partial (10%) "
TC16721	"weakly similar to pre-mRNA splicing SRp75 - human, partial (32%) "
TC6881	"similar to Similar to chromobox homolog 1 (Drosophila HP1 beta) {Danio rerio}, partial (53%)
TC16140	"weakly similar to SD06874p {Drosophila melanogaster}, partial (6%) "
TC7484	"weakly similar to Similar to chaperonin containing TCP1 subunit 8 (theta) {Xenopus laevis}, partial (60%) "
TC16605	"similar to hypothetical protein {Plasmodium falciparum 3D7}, partial (0%) "
TC11576	"ribosomal protein L37 {Schistosoma mansoni}, complete"
TC10529	"similar to Probable dynein light chain 1 cytoplasmic. {Caenorhabditis elegans}, partial (95%) "
TC8614	"similar to RE07451p {Drosophila melanogaster}, partial (81%) "
TC7314	"homologue to cytochrome c oxidase subunit 1 {Schistosoma mansoni}, partial (28%) "
TC8560	"weakly similar to putative 60S RIBOSOMAL PROTEIN L36 {Oryza sativa (japonica cultivar-group)}, partial (58%) "
TC10547	"homologue to ribosomal protein S8 {Schistosoma japonicum}, complete"
TC16630	"similar to protein T6D22.2 [imported] - Arabidopsis thaliana, partial (16%) "
TC17564	"similar to Unknown (protein for MGC:56459) {Danio rerio}, partial (67%) "
TC11170	"similar to Usmg5 protein {Mus musculus}, partial (31%) "
TC17090	"weakly similar to 40S ribosomal protein S21 {Ictalurus punctatus}, partial (54%) "
TC16972	"similar to phosphate carrier protein precursor mitochondrial splice form B - bovine, partial (81%) "
TC17388	"similar to ribosomal protein S15a {Taenia solium}, complete"
TC16739	"14-3-3 protein homolog 1. [Blood fluke], partial (31%) "
TC7713	"similar to ribosomal protein S14 - mouse, partial (88%) "
TC16732	"weakly similar to DnaJ-like protein {Cercopithecus aethiops}, partial (76%) "
TC10736	"homologue to similar to GenBank Accession Number AJ312339 putative ribosomal protein L27A protein in, complete"
TC16803	"weakly similar to ribosomal protein L6 {Ictalurus punctatus}, partial (49%) "
TC7475	"similar to 60S ribosomal protein L8. [Rat], partial (97%) "
TC11590	"ubiquitin/ribosomal fusion protein {Schistosoma japonicum}, complete"
TC7403	"similar to 60S ribosomal protein L30. [Amphoxius], partial (92%) "
TC16897	"similar to Unknown (protein for MGC:11850) {Mus musculus}, partial (25%) "
TC7478	"similar to ribosomal protein S25 {Branchiostoma belcheri}, partial (72%) "
TC11327	"weakly similar to 40S ribosomal protein S12. {Cyanophora paradoxa}, partial (72%) "
TC6874	"similar to GM02242p {Drosophila melanogaster}, partial (72%) "
TC7041	"weakly similar to ribosomal protein L7a cytosolic - chicken, partial (86%) "
TC17430	
TC7437	"weakly similar to cisplatin resistance-associated overexpressed protein {Homo sapiens}, partial (19%) "

CLUSTER 2

TC10486	"Glutathione S-transferase 28 kDa (EC 2.5.1.18) (GST 28) (SM28 antigen) (Protective 28 kDa antigen), complete"
TC13533	"homologue to similar to GenBank Accession Number AY072287 ribosomal protein L3 Schistosoma japonicum}, complete"
TC13852	"similar to ribosomal protein S18 {Branchiostoma belcheri}, partial (92%) "
TC10912	"similar to ribosomal protein L17A - fruit fly (Drosophila melanogaster), partial (97%) "
TC16808	"weakly similar to ribosome-associated protein P40 {Bombyx mori}, partial (59%) "
TC11169	"similar to Usmg5 protein {Mus musculus}, partial (41%) "
TC17069	"weakly similar to 60S ribosomal protein L35 {Euphorbia esula}, partial (47%) "
TC13971	"Glutathione S-transferase 26 kDa (EC 2.5.1.18) (GST 26) (SM26/2 antigen) (GST class-alpha)., partial (94%) "
TC14946	
TC16078	"similar to 60S ribosomal protein L11. [Rat], partial (76%) "
TC11413	"similar to nucleoside diphosphate kinase {Schistosoma japonicum}, partial (94%) "
TC13523	"homologue to similar to GenBank Accession Number S41224 hnRNP protein in Xenopus laevis {Schistosoma japonicum}, partial (36%) "
TC7098	"similar to 40S ribosomal protein S24. [Japanese pufferfish Takifugu rubripes], partial (78%) "
TC7444	
TC13573	"23 kDa integral membrane protein (SM23). [Blood fluke], complete"
TC13698	"40S rRNA protein homolog {Schistosoma mansoni}, complete"
TC16743	"CG11624-PA {Drosophila melanogaster}, partial (33%) "
TC10432	"similar to CG3395-PA {Drosophila melanogaster}, partial (92%) "
TC7295	
TC16643	"homologue to elongation factor 1-alpha {Schistosoma mansoni}, partial (40%) "
TC17433	"homologue to similar to GenBank Accession Number X71081 ribosomal protein S8 in Xenopus laevis, complete"
TC13607	"similar to Similar to ribosomal protein L13a {Xenopus laevis}, partial (61%) "
TC8131	"similar to 40S ribosomal protein S11. [Rat], partial (74%) "

TC7643 "weakly similar to ribosomal protein L14 {Spodoptera frugiperda}, partial (35%) "
TC10528 "Probable dynein light chain (T-cell-stimulating antigen SM10). [Blood fluke], complete"
TC10700 "similar to C. elegans RPS-26 protein (corresponding sequence F39B2.6) {Caenorhabditis elegans}, partial (79%) "
TC16547 "similar to heat shock protein 90 {Dendronephthya klunzingeri}, partial (83%) "
TC14141 "weakly similar to similar to GenBank Accession Number Z98600 cofilin in Schizosaccharomyces pombe, partial (32%) "
TC7377 "similar to Y-box binding protein {Schistosoma mansoni}, partial (46%) "
TC17080 "weakly similar to ribosomal protein L18 {Petromyzon marinus}, complete"
TC10506 "homologue to similar to GenBank Accession Number L78668 60S ribosomal protein L5A {Schistosoma japonicum}, complete"
TC7748 "weakly similar to ribosomal protein L21 {Argopecten irradians}, partial (89%) "
TC17017 "homologue to hypothetical protein {Schistosoma japonicum}, complete"
TC17261 "weakly similar to hypothetical protein T13H5.2 - Caenorhabditis elegans, partial (14%) "
TC10617 "similar to ubiquitin {Ciona savignyi}, partial (49%) "
TC16537 "Heat shock 70 kDa homolog protein (HSP70) (Major surface antigen). [Blood fluke], complete"
TC10668 "similar to similar to NM_058275 probable 60S ribosomal protein L7 in Caenorhabditis elegans, partial (95%) "
TC7421 "similar to Cs1 protein {Schistosoma japonicum}, partial (82%) "
TC16827 "homologue to similar to NM_006276 splicing factor arginine/serine-rich 7 {Schistosoma japonicum}, partial (70%) "
TC17770 "phosphatidylinositol-4-phosphate 5-kinase putative {Plasmodium falciparum 3D7}, partial (0%)
TC7096 "homologue to similar to GenBank Accession Number U30454 ribosomal protein S2 in Urechis caupo, partial (93%) "
TC10570 "weakly similar to hypothetical protein putative Profilin/allergen {Schistosoma japonicum}, partial (29%) "
TC17455 "similar to GrpE protein homolog mitochondrial precursor. [Fission yeast], partial (8%) "
TC8574 "similar to Small nuclear ribonucleoprotein SM D3 (snRNP core protein D3) (SM- D3). [Fruit fly], partial (55%) "
TC16552 "homologue to heat shock protein 86 - fluke (Schistosoma mansoni) (fragment), partial (53%) "
TC13511 "homologue to AUT1 {Schistosoma mansoni}, partial (46%) "
TC7420 "similar to Cs1 protein {Schistosoma japonicum}, partial (96%) "
TC10452 "homologue to similar to NM_078658 40S ribosomal protein S5 {Schistosoma japonicum}, partial (92%) "
TC10473 "weakly similar to SET protein (HLA-DR associated protein II) (PHAPII) (Phosphatase 2A inhibitor I2PP2A). [Human], partial (68%) "
TC10682 "weakly similar to RE30690p {Drosophila melanogaster}, partial (82%) "
TC8189 "similar to Dynein 8 kDa light chain flagellar outer arm. {Chlamydomonas reinhardtii}, partial (96%) "
TC11430 "thioredoxin {Schistosoma mansoni}, complete"
TC16738 "14-3-3 protein homolog 1. [Blood fluke], complete"
TC6863 "homologue to 14-3-3 protein homolog 1. [Blood fluke], partial (27%) "
TC16806 "similar to NADH dehydrogenase 6 {Schistosoma mansoni}, complete"
TC17271 "similar to Cd151 protein {Mus musculus}, partial (6%) "
TC16638 "weakly similar to protein T6D22.2 [imported] - Arabidopsis thaliana, partial (15%) "
TC16731 "weakly similar to DnaJ homolog subfamily A member 4 (MmDjA4). [Mouse], partial (33%) "
TC16794
TC10691 "thioredoxin peroxidase {Schistosoma mansoni}, complete"
TC16584 "weakly similar to Ubiquitin-activating enzyme E1 1 (Poly(A)+ RNA transport protein 3). [Fission yeast], partial (4%) "
TC14369 "similar to Small nuclear ribonucleoprotein F (snRNP-F) (Sm protein F) (Sm-F) (SmF). [Mouse], partial (79%) "
TC7615 "similar to ATP synthase lipid-binding protein-like protein {Schistosoma japonicum}, complete"
TC11552 "homologue to cytochrome b {Schistosoma mansoni}, complete"
TC16792 "leucine-rich protein {Schistosoma mansoni}, complete"
TC7432
TC17148
TC8215 "weakly similar to CG7939-PC {Drosophila melanogaster}, partial (91%) "
TC10484 "homologue to Actin. [Pacific oyster], partial (18%) "
TC13521 "homologue to similar to GenBank Accession Number S41224 hnRNP protein in Xenopus laevis {Schistosoma japonicum}, partial (92%) "
TC6872 "similar to tubulin alpha chain - sea urchin (Paracentrotus lividus), partial (97%) "
TC18131 "60S ribosomal protein L37a (Fragment). [Blood fluke], complete"
TC14109 "homologue to similar to GenBank Accession Number AF004672 ribosomal protein L41 in Phaffia rhodozyma, complete"
TC16627 "Actin 2. [Blood fluke], partial (45%) "
TC16631 "homologue to elongation factor 1-alpha {Schistosoma mansoni}, complete"
TC10435 "similar to CG3395-PA {Drosophila melanogaster}, partial (86%) "
TC10483 "Actin 1. [Blood fluke], partial (50%) "
TC7447 "similar to Similar to ribosomal protein L18a {Xenopus laevis}, partial (94%) "
TC16645 "similar to elongation factor 1-alpha {Schistosoma mansoni}, partial (22%) "
TC6902 "weakly similar to Similar to ribosomal protein L10a {Xenopus laevis}, partial (58%) "
TC7040 "weakly similar to ribosomal protein L26 {Ictalurus punctatus}, partial (97%) "

CLUSTER 3

TC10509
TC19220 "weakly similar to Unknown (protein for MGC:20781) {Homo sapiens}, partial (11%) "
TC13775 "Antigen SM21.7. [Blood fluke], complete"
TC16807 "homologue to NADH dehydrogenase 6 {Schistosoma mansoni}, complete"
TC14303 "homologue to ATPase subunit 6 {Schistosoma mansoni}, complete"
TC17303
TC11510
TC17477 "homologue to NADH dehydrogenase subunit 2 {Schistosoma mansoni}, complete"

TC16858	"similar to ADP/ATP carrier {Trypanosoma brucei brucei}, partial (57%) "
TC18675	"similar to similar to alpha-L-fucosidase {Schistosoma japonicum}, partial (31%) "
TC7477	"similar to pyruvate phosphate dikinase (pyruvate orthophosphate dikinase) [imported] - Agrobacterium, partial (1%) "
TC16782	"Enolase (EC 4.2.1.11) (2-phosphoglycerate dehydratase) (2-phospho-D- glycerate hydro-lyase)., complete"
TC11243	"similar to hypothetical protein {Plasmodium falciparum 3D7}, partial (5%) "
TC13475	"similar to JF-2 {Schistosoma japonicum}, partial (67%) "
TC9568	"similar to 60S ribosomal protein L13a (23 kDa highly basic protein). [Human], partial (77%) "
TC15827	"homologue to Similar to tubulin alpha 1 {Xenopus laevis}, partial (24%) "
TC10468	
TC7792	"similar to PP2A inhibitor {Tetraodon fluviatilis}, partial (59%) "
TC16557	"myosin heavy chain - fluke (Schistosoma mansoni) (strain Brazilian LE), complete"
TC17193	"similar to NADH dehydrogenase subunit 4 {Schistosoma mansoni}, partial (26%) "
TC12604	"similar to hypothetical protein {Plasmodium falciparum 3D7}, partial (1%) "
TC8122	"homologue to unnamed protein product {Homo sapiens}, partial (6%) "
TC8149	
TC10480	"elastase {Schistosoma mansoni}, partial (41%) "
TC13518	"homologue to NADH dehydrogenase subunit 1 {Schistosoma mansoni}, partial (96%) "
TC16747	"weakly similar to Hypothetical protein Y69A2AR.18a {Caenorhabditis elegans}, partial (61%) "
TC8436	
TC12356	
TC14659	"similar to RNA binding motif protein 8A {Homo sapiens}, partial (72%) "
TC7207	"similar to Tubulin alpha chain. [Eastern newt Triturus viridescens], partial (26%) "
TC13722	"homologue to NADH dehydrogenase subunit 5 {Schistosoma mansoni}, complete"
TC11778	
TC7180	"homologue to similar to Dictyostelium discoideum (Slime mold). MYOM protein, partial (1%) "
TC17934	"similar to hypothetical protein {Plasmodium falciparum 3D7}, partial (0%) "
TC10090	
TC8089	"weakly similar to Pyruvate kinase M2 isozyme (EC 2.7.1.40). [Rabbit], partial (23%) "
TC10872	"weakly similar to similar to HMG1-like protein - fruit fly {Schistosoma japonicum}, partial (85%) "
TC7584	
TC17147	"similar to similar to human carbonyl reductase (NADPH) PIR Accession Number A61271; Method: conceptual, partial (55%) "
TC18149	
TC17016	"stathmin-like protein {Schistosoma mansoni}, complete"
TC7095	
TC7310	"homologue to cytochrome c oxidase subunit 1 {Schistosoma mansoni}, complete"
TC14660	"weakly similar to CG8781-PA {Drosophila melanogaster}, partial (86%) "
TC18151	"weakly similar to Unknown (protein for MGC:41267) {Mus musculus}, partial (84%) "
TC8661	"weakly similar to DC6 {Homo sapiens}, partial (71%) "
TC8910	"Tubulin beta-2 chain. [Fruit fly], partial (28%) "
TC14564	"homologue to Similar to adenosine kinase {Danio rerio}, partial (6%) "
TC17284	"similar to ATP synthase oligomycin sensitivity conferral protein mitochondrial precursor (EC 3.6.3.14) (OSCP)., partial (27%) "
TC7850	"similar to actin-filament fragmenting protein {Echinococcus granulosus}, partial (36%) "
TC7158	"similar to Fibrillarin. [Fission yeast], partial (13%) "
TC16860	"similar to ADP ATP carrier protein F20O9.60 - Arabidopsis thaliana, partial (16%) "
TC7358	"similar to Importin alpha-3 subunit (Karyopherin alpha-3 subunit) (SRP1-gamma). [Human], partial (84%) "
TC12249	"weakly similar to Hypothetical protein C33H5.19 {Caenorhabditis elegans}, partial (16%) "

CLUSTER 4

TC11102	"similar to hypothetical protein F19B6.1a - Caenorhabditis elegans, partial (4%) "
TC14000	"similar to TPBF gene product {Acanthamoeba castellanii}, partial (4%) "
TC14763	"homologue to ribosomal protein S25 putative {Plasmodium falciparum 3D7}, partial (8%) "
TC13632	"unknown {Schistosoma mansoni}, complete"
TC13719	
TC14617	
TC17104	"weakly similar to tegumental protein {Schistosoma japonicum}, partial (42%) "
TC13565	"myosin light chain {Schistosoma mansoni}, complete"
TC13726	
TC13534	"similar to unknown {Schistosoma mansoni}, partial (75%) "
TC14349	"similar to similar to XM_082517 CG9399 gene product in Drosophila melanogaster {Schistosoma japonicum}, partial (94%) "
TC13727	"homologue to gag-pol polyprotein {Clonorchis sinensis}, partial (1%) "
TC7800	"similar to R07E5.13 protein (clone R07E5) - Caenorhabditis elegans, partial (45%) "
TC16878	"similar to Glycerol-3-phosphate dehydrogenase [NAD+] cytoplasmic (EC 1.1.1.8) (GPD-C) (GPDH-C)., partial (50%) "
TC16751	"homologue to similar to GenBank Accession Number AY044241 glutamine synthetase {Schistosoma japonicum}, complete"
TC17004	"homologue to similar to cytochrome c {Schistosoma japonicum}, complete"
TC10620	
TC16870	"ATP:guanidino kinase SMC74 (EC 2.7.3.-) (ATP:guanidino phosphotransferase). [Blood fluke], partial (96%) "
TC8186	
TC13516	
TC8250	
TC12523	"similar to NADH dehydrogenase subunit 4 {Schistosoma mansoni}, partial (36%) "

TC8935	"homologue to unknown {Homo sapiens}, partial (11%) "
TC17192	"homologue to NADH dehydrogenase subunit 4 {Schistosoma mansoni}, complete"
TC8821	"weakly similar to synapse associated protein 1 {Mus musculus}, partial (15%) "
TC7036	"similar to CCAAT-box DNA binding protein subunit B {Plasmodium falciparum}, partial (2%)

CLUSTER 5

TC19377	
TC13507	"similar to hypothetical protein Y66H1A.4 - Caenorhabditis elegans, partial (26%) "
TC16575	"major egg antigen {Schistosoma mansoni}, partial (21%) "
TC10348	
TC11145	
TC8654	"similar to Anti-Mullerian hormone type II receptor precursor (EC 2.7.1.37) (AMH type II receptor), partial (3%) "
TC10002	
TC13584	"oj991113_30.13 {Oryza sativa (japonica cultivar-group)}, partial (2%) "
TC6924	"similar to hypothetical protein {Macaca fascicularis}, partial (13%) "
TC14289	
TC15088	"similar to KIAA0228 protein {Homo sapiens}, partial (16%) "
TC13882	"similar to 26S proteasome regulatory subunit {Dermacentor variabilis}, partial (94%) "
TC14265	"weakly similar to symbol=BG:DS00797.1; cDNA=method:"sim4" score:"1000.0" desc:"LD32761 LD Drosophila, partial (54%) "
TC13335	
TC18006	"weakly similar to Hypothetical protein Y65B4A.3 {Caenorhabditis elegans}, partial (31%) "
TC15206	"similar to DEAD-box protein abstrakt. [Fruit fly], partial (12%) "
TC12788	
TC14062	"homologue to similar to GenBank Accession Number AF063024 phosphate transporter 1 in Cricetulus griseus, partial (92%) "
TC18377	"weakly similar to gamma-filamin {Homo sapiens}, partial (2%) "
TC10246	
TC11847	"weakly similar to unnamed protein product {Homo sapiens}, partial (21%) "
TC8528	"weakly similar to UGNT {Takifugu rubripes}, partial (17%) "
TC14726	"homologue to similar to Plasmodium falciparum (isolate 3D7). Hypothetical protein {Dictyostelium discoideum}, partial (2%) "
TC16965	"weakly similar to Glucosidase II {Homo sapiens}, partial (42%) "
TC8548	"similar to unnamed protein product {Mus musculus}, partial (10%) "
TC12261	"weakly similar to probable lipolytic enzyme PA5384 [imported] - Pseudomonas aeruginosa (strain PAO1), partial (22%) "
TC13485	
TC16610	"similar to Hypothetical protein R193.3 {Caenorhabditis elegans}, partial (4%) "
TC16194	"similar to special lobe-specific silk protein ssp160A {Chironomus pallidivittatus}, partial (1%) "
TC12548	
TC18339	"weakly similar to Similar to hypothetical protein DKFZp434B227 {Mus musculus}, partial (10%) "
TC7340	"homologue to This is the longest open reading frame of the sequence that starts with Met {Schistosoma mansoni}, complete"
TC10975	"similar to unnamed protein product {Homo sapiens}, partial (16%) "
TC10950	"weakly similar to carboxypeptidase gp180 - Anas sp., partial (6%) "
TC12139	"weakly similar to Alanyl-tRNA synthetase (EC 6.1.1.7) (Alanine--tRNA ligase) (AlaRS). [Human], partial (19%) "
TC18747	"similar to hypothetical protein {Schistosoma japonicum}, partial (91%) "
TC13659	"weakly similar to hypothetical protein {Plasmodium falciparum 3D7}, partial (2%) "
TC9257	
TC15937	
TC16854	"weakly similar to homologue of the murine Llglh gene {Homo sapiens}, partial (3%) "
TC17255	"similar to hypothetical protein with Src homology 3, partial (54%) "
TC18001	
TC7523	"similar to hypothetical protein {Schistosoma japonicum}, partial (93%) "
TC7710	"weakly similar to beta-1 2-N-acetylglucosaminyltransferase II {Sus scrofa}, partial (17%) "
TC13646	"similar to adaptor protein DAPP1 {Mus musculus}, partial (5%) "
TC17475	"homologue to hypothetical protein conserved {Plasmodium falciparum 3D7}, partial (6%) "
TC11505	
TC13799	
TC7884	"similar to similar to GenBank Accession Number AJ291614 actin related protein 2/3 protein complex subunit p16, complete"
TC15967	"similar to similar to GenBank Accession Number BC013428 PP1201 protein in Homo sapiens {Schistosoma japonicum}, partial (64%) "
TC19691	
TC19645	
TC18460	
TC19414	

CLUSTER 6

TC12502	"similar to hemochromatosis gene product HFE splice variant delE2 {Rattus norvegicus}, partial (5%) "
TC14160	
TC13169	"similar to hypothetical protein {Pseudomonas syringae pv. tomato str. DC3000}, partial (17%) "
TC15581	"weakly similar to Dynamin-1 (EC 3.6.1.50) (Dynamin BREDNM19). [Mouse], partial (6%) "
TC11923	
TC15484	"similar to hypothetical protein C09H6.a {Caenorhabditis elegans}, partial (9%) "

TC12198	"similar to mucin-like protein - Arabidopsis thaliana, partial (4%) "
TC8239	
TC10897	"homologue to unknown {Schistosoma mansoni}, complete"
TC12085	
TC16345	"homologue to Unknown (protein for MGC:16752) {Homo sapiens}, partial (5%) "
TC13351	"weakly similar to Similar to LOC132671 {Mus musculus}, partial (10%) "
TC14589	"similar to conserved hypothetical protein {Xanthomonas campestris pv. campestris str. ATCC 33913}, partial (3%) "
TC16176	
TC15161	
TC12228	"weakly similar to unknown {Homo sapiens}, partial (36%) "
TC14749	"weakly similar to Lor protein {Mus musculus}, partial (8%) "
TC12797	"weakly similar to probable membrane transport protein STY3643 [imported] - Salmonella enterica subsp. enterica, partial (15%) "
TC8196	
TC13487	"homologue to Eggshell protein precursor (Chorion protein). [Blood fluke], partial (55%) "
TC14235	"weakly similar to cytochrome b5 {Ciona savignyi}, partial (54%) "
TC8804	"weakly similar to retinoid X receptor RXR-2 {Schistosoma mansoni}, partial (10%) "
TC7011	"similar to Putative retroelement {Oryza sativa} [Oryza sativa (japonica cultivar-group)], TC10861 "similar to hypothetical protein {Bacillus megaterium}, complete"
TC12944	
TC7536	"weakly similar to DjVLGA {Dugesia japonica}, partial (22%) "
TC12926	
TC7139	"weakly similar to unnamed protein product {Homo sapiens}, partial (7%) "
TC11241	"similar to EST gb|ATTS1136 comes from this gene. {Arabidopsis thaliana}, partial (6%) "
TC18160	
TC11291	"homologue to unnamed protein product {Mus musculus}, partial (8%) "
TC8175	"similar to EsV-1-103 {Ectocarpus siliculosus virus}, partial (3%) "
TC11826	"similar to similar to GenBank Accession Number AY070592 RE69393p in Drosophila melanogaster, partial (76%) "
TC14750	
TC18406	"weakly similar to Stage V sporulation protein AD {Bacillus cereus ATCC 14579}, partial (10%) "
TC11986	"similar to HPHase {Homo sapiens}, partial (8%) "
TC11472	
TC17501	"weakly similar to Beclin 1-like protein. [Fruit fly], partial (21%) "
TC7649	"similar to ras-related GTPase rag splice form A [imported] - human, partial (69%) "
TC8007	"similar to histone acetyltransferase Gcn5 putative {Plasmodium falciparum 3D7}, partial (0%) "
TC7019	
TC7872	"weakly similar to Probable RNA 3'-terminal phosphate cyclase (EC 6.5.1.4) (RNA-3'- phosphate cyclase) (RNA cyclase)., partial (16%) "
TC10194	
TC10450	"weakly similar to unnamed protein product {Mus musculus}, partial (16%) "
TC12567	
TC13457	"Eggshell protein precursor (Chorion protein). [Blood fluke], complete"
TC15060	
TC11362	"similar to ankyrin G119 {Homo sapiens}, partial (5%) "
TC18166	
TC12020	"similar to serine/threonine protein kinase (putative) {Lactobacillus plantarum WCFS1}, partial (2%) "
TC7509	
TC13084	
TC17669	"unknown {Arabidopsis thaliana}, partial (20%) "
TC18760	
TC13492	"homologue to eggshell precursor protein {Schistosoma mansoni}, partial (26%) "
TC17000	
TC16777	"homologue to Extracellular superoxide dismutase [Cu-Zn] precursor (EC 1.15.1.1) (EC-SOD). [Blood fluke], partial (95%) "
TC16781	"Extracellular superoxide dismutase [Cu-Zn] precursor (EC 1.15.1.1) (EC-SOD). [Blood fluke], partial (35%) "
TC10637	"weakly similar to Aquaporin 9 (Small solute channel 1). [Human], partial (20%) "
TC8004	"weakly similar to hypothetical protein {Shewanella oneidensis MR-1}, partial (15%) "
TC9163	"similar to Serine/threonine-protein kinase unc-51 (EC 2.7.1.-) (Uncoordinated protein 51)., partial (8%) "
TC11502	
TC11963	
TC16762	
TC7005	"similar to putative senescence-associated protein {Pisum sativum}, partial (16%) "
TC11224	"unknown {Schistosoma mansoni}, complete"
TC6945	
TC12088	
TC18371	
TC9012	
TC18019	
TC10526	"LGG {Schistosoma mansoni}, complete"
TC18790	"similar to transport protein [imported] - Sulfolobus solfataricus, partial (3%) "
TC10493	"cathepsin B1 isotype 1 {Schistosoma mansoni}, complete"
TC18412	"homologue to hypothetical protein FLJ10504 {Homo sapiens}, partial (3%) "

TC16817	
TC18007	
TC19260	"similar to Unknown protein {Arabidopsis thaliana}, partial (10%) "
TC18066	
TC14869	
TC12296	"weakly similar to Laminin related protein 1 {Caenorhabditis elegans}, partial (2%) "
TC8129	"similar to beta-1 4-galactosyltransferase {Gallus gallus}, partial (4%) "
TC14899	
TC7105	"similar to lic2 product {Haemophilus influenzae}, partial (69%) "
TC10898	"homologue to unknown {Schistosoma mansoni}, partial (72%) "
TC18469	"similar to HPHase {Homo sapiens}, partial (8%) "
TC9312	
TC16448	
TC19297	
TC19737	"similar to unknown protein {Arabidopsis thaliana}, partial (3%) "
TC9870	"similar to Probable glutaminyl-tRNA synthetase (EC 6.1.1.18) (Glutamine--tRNA ligase) (GlnRS)., partial (10%) "
TC16289	
TC14156	
TC19434	
TC13658	"histone H3 - chicken, complete"
TC19412	
TC9371	
TC10864	"weakly similar to putative ARM-1 protein {Gallus gallus}, partial (17%) "
TC7206	"similar to extensin homolog T9E8.80 - Arabidopsis thaliana, partial (2%) "
TC17058	
TC17188	"similar to hypothetical protein all7010 [imported] - Nostoc sp. (strain PCC 7120) plasmid pCC7120alpha, partial (3%) "
TC17189	
TC18655	"similar to unnamed protein product {Homo sapiens}, partial (6%) "
TC7194	
TC7563	"similar to polyprotein {Schistosoma japonicum}, partial (7%) "
TC8997	
