# Supplementary material for: Gene expression patterns during adaptation of a helminth parasite to different environmental niches
Source: Genome Biol. 2007 Apr 24;8(4):R65. doi: 10.1186/gb-2007-8-4-r65 (PMC1896014; doi:10.1186/gb-2007-8-4-r65)
Supplement: Additional data file 6 — Transcript comparisons are between sporocysts (Sp) and cercariae (Ce), and adult worms (Ad) and cercariae [file gb-2007-8-4-r65-S6.pdf]

NAME	NOTES	Sp/Ce	Sp/Ce	Ad/Ce	Ad/Ce
TC16981	retinoid X receptor RXR-2 {Schistosoma mansoni}, complete	2.63	3.42	0.69	0.99
TC17002	similar to Thyroid hormone receptor-associated protein complex 150 kDa component (Trap150). [Human], partial (3%)	1.89	1.80	0.68	-0.13
TC14740	similar to similar to NM_015344 brain my047 protein leptin receptor overlapping transcript-like 1, partial (90%)	1.01	1.91	0.57	0.46
TC7362	receptor for activated PKC {Schistosoma mansoni}, complete	2.52	3.30	0.35	0.50
TC7363	receptor for activated PKC {Schistosoma mansoni}, partial (60%)	2.84	3.35	0.45	0.68
TC17556	weakly similar to nuclear receptor binding factor-1 {Rattus norvegicus}, partial (19%)	1.51	1.59	0.12	0.44
TC16901	homologue to trispanning orphan receptor; TORE {Schistosoma mansoni}, partial (9%)	0.44	1.32	-0.03	0.12
TC17142	similar to vomeronasal receptor V1RH14 {Mus musculus}, partial (8%)	0.48	2.83	0.36	1.32
TC7994	weakly similar to Thyroid receptor interacting protein 12 (TRIP12). [Human], partial (10%)	0.20	0.71	1.40	1.49
TC8654	similar to Anti-Mullerian hormone type II receptor precursor (EC 2.7.1.37) (AMH type II receptor), partial (3%)	0.01	1.34	3.08	3.16
TC8804	weakly similar to retinoid X receptor RXR-2 {Schistosoma mansoni}, partial (10%)	-0.37	0.42	6.42	6.84
TC8870	homologue to Epidermal growth factor receptor kinase substrate EPS8. [Human], partial (1%)	-0.14	-0.24	0.84	1.02
TC16836	similar to scavenger receptor protein CI precursor {Drosophila melanogaster}, partial (3%)	-0.15	-0.19	1.10	1.47
TC16107	similar to seven transmembrane-domain receptor {Homo sapiens}, partial (1%)	0.08	0.42	1.45	1.40
TC19011	similar to novel immune-type receptor 4.1 {Danio rerio}, partial (4%)	0.37	1.99	-0.27	-0.27
TC14923	insulin receptor protein kinase RTK-2 {Schistosoma mansoni}, partial (75%)	0.03	1.06	-0.58	-0.50
TC13571	weakly similar to Thyroid hormone receptor-associated protein complex 150 kDa component (Trap150). [Human], partial (4%)	0.59	1.37	0.17	-0.29
TC11533	weakly similar to nuclear receptor binding factor-like protein {Arabidopsis thaliana}, partial (11%)	1.32	2.13	-0.68	-0.28
TC7504	similar to trace amine receptor 1 {Macaca mulatta}, partial (4%)	0.86	0.94	-0.20	-0.22
TC17448	similar to TNF-receptor-associated factor 1 {Drosophila melanogaster}, partial (4%)	1.10	1.91	-0.01	-0.07
TC16964	weakly similar to Peripheral-type benzodiazepine receptor (PBR) (PKBS) (Mitochondrial benzodiazepine receptor)., partial (21%)	2.40	3.13	-0.02	0.04
TC17447	weakly similar to Similar to serine/threonine kinase receptor associated protein {Danio rerio}, partial (34%)	1.34	1.42	-0.32	-0.16
